# Supplementary material for: Comparing Seamounts and Coral Reefs with eDNA and BRUVS Reveals Oases and Refuges on Shallow Seamounts
Source: Biology (Basel). 2023 Nov 17;12(11):1446. doi: 10.3390/biology12111446 (PMC10669620; doi:10.3390/biology12111446)
Supplement: Supplementary file 1 [file biology-12-01446-s001.zip › biology-2662398-supplementary.pdf]

Supplementary Materials: Environmental DNA and Video Surveys of Seamounts and Coral Reefs Reveal Biomass Oases on Shallow Seamounts

Supplementary Section S1: General data description

Table S1: BRUVS and eDNA samples collected on 5 different environments: coral reefs (15 m), seamounts (50 m), continental slopes (150 m), seamounts (250 m) and seamounts (500 m).

Environment	Site name	Latitude	Longitude	Summit depth (m)	Summit height (m)	eDNA		BRUVS	
						Sample size	Sample depth range (m)	Sample size	Sample depth range (m)
Coral reef	Noumea	-22.29341	166.2149	-	-	6	10.6 - 14.3	13	8.8 - 14
	St Vincent	-22.05113	165.9717	-	-	6	10.9 - 13.9	14	5.5 - 15
	Bourail	-21.68457	165.4693	-	-	6	2.5 - 19.5	15	1.9 - 23.4
	Nepoui	-21.39059	164.928	-	-	6	4.6 - 18.9	16	3.2 - 19.8
	Gatope	-20.9797	164.5894	-	-	6	6.7 - 19.7	15	4.4 - 22
	Poum	-20.36597	163.9297	-	-	6	7.7 - 16.6	16	7.1 - 20
	Grand Lagon Nord	-19.41589	163.2245	-	-	6	20 - 22.5	14	18 - 28
Seamount_50	Torche	-22.87503	167.6631	45	1318	10	45 - 58	5	47 - 62
	Antigonia	-23.42824	168.0752	54	1330	10	54 - 70	8	56 - 66
	Capel	-25.03758	159.5323	60	3054	10	60 - 70	10	65 - 69
	Fairway	-21.04964	162.255	62	2964	10	62 - 67	10	63 - 67
Deep slope_150	St Vincent	-22.12531	166.0376	-	-	10	80 - 219	7	120 - 180
	Nepoui	-21.42488	164.9774	-	-	10	88 - 218	10	105 - 150
	Poum	-20.15007	163.7853	-	-	10	100 - 185	8	100 - 220
	Grand Lagon Nord	-19.45239	163.2159	-	-	10	85 - 235	10	118 - 150
Seamount_250	Crypthelia	-23.3078	168.2498	195	1627	10	201 - 236	9	200 - 244
	Kaimon Maru	-24.74137	168.1411	236	1799	10	238 - 325	8	238 - 340
	Jumeau Ouest	-23.68215	168.0081	239	1119	10	242 - 313	8	245 - 339
	Argo	-23.09286	159.463	299	2251	10	299 - 313	8	301 - 312
Seamount_500	Stylaster	-23.6461	167.7134	434	801	10	439 - 488	8	444 - 491
	Ile Des Pins	-22.38325	167.407	470	818	10	469 - 488	5	480 - 506
	Eponge	-24.91183	168.363	511	1932	10	518 - 570	7	520 - 552

Table S2: Environmental variables used in the BRT modelling of species richness, fish biomass and large predator biomass for BRUVS, and eDNA MOTU richness. BRUVS micro-habitat variables were extracted through a semi-quantitative scale. Micro-habitat covers were evaluated on a 0-100% at a 5% precision. Substrate complexity and topography were evaluated on a scale of 0 to 5 and transformed in percentages. These variables were then used to calculate the Shannon diversity indices used in the boosted regression tree modelling.

Variable name	Description	Use in BRTs	Reference	Source
Depth	Depth value taken on the ship's echo sounder	Used	(Priede et al. 2013; Letessier et al. 2019)	Ship
Habitat diversity (BRUVS only)	Shannon diversity indice on percentages of visually evaluated habitat features (Bare substrate, vegetation, filter feeders, coral, indetermined, sand, gravel, rubbles, rocks, reef structure, substrate complexity, topography)	Used	(Eduardo et al. 2018; Sih et al. 2019)	BRUVS still images
Turbidity	Visually evaluated in four categories (Clear, Particulate, Turbid, Very turbid)	Used	(Baletaud et al. 2022)	BRUVS still images
Travel time	Time to travel from Noumea's center using the shortest path	Used	(Maire et al. 2016)	(Maire et al. 2016)
Chl-a concentration	Chlorophyll concentration in milligram.m3	Removed (correlated)	(Leitner et al. 2020)	https://resources.marine.copernicus.eu/product-detail/OCEANCOLOUR_GLO_CHL_L4_REP_OBSERVATIONS_009_082/INFORMATION
Seafloor temperature	Seafloor potential temperature (degree C)	Used	(Tittensor et al. 2010; Woolley et al. 2016)	https://resources.marine.copernicus.eu/product-detail/GLOBAL_REANALYSIS_PHY_001_030/INFORMATION
Minimal SST	Average sea surface temperature of the coldest month of each year over the last 10 years (degrees C).	Removed (correlated)	(Tittensor et al. 2010)	https://podaac.jpl.nasa.gov/dataset/MUR-JPL-L4-GLOB-v4.1

Mean SST	Average temperature over the last 10 years (degrees C)	Used	(Tittensor et al. 2010)	https://podaac.jpl.nasa.gov/dataset/MUR-JPL-L4-GLOB-v4.1
Maximal SST	Average temperature of the hottest month of each year over the last 10 years (degrees C).	Removed (correlated)	(Tittensor et al. 2010)	https://podaac.jpl.nasa.gov/dataset/MUR-JPL-L4-GLOB-v4.1
Suspended particulate matter	Inorganic suspended particulate matter (g/m3)	Removed (correlated)	(Capblancq 1990)	https://resources.marine.copernicus.eu/product-download/OCEANCOLOUR_GLO_OPTICS_L4_REP_OBSERVATIONS_009_081
Salinity	Salinity (10^-3)	Removed (correlated)	(Barletta et al. 2005)	https://resources.marine.copernicus.eu/product-detail/GLOBAL_REANALYSIS_PHY_001_030/INFORMATION
Eastward velocity	Eastward velocity (m.s-1)	Used	(Rogers 2018)	https://resources.marine.copernicus.eu/product-detail/GLOBAL_REANALYSIS_PHY_001_030/INFORMATION
Northward velocity	Northward velocity (m.s-1)	Used	(Rogers 2018)	https://resources.marine.copernicus.eu/product-detail/GLOBAL_REANALYSIS_PHY_001_030/INFORMATION
Environnemental stratum	Coral reef, continental slope (150 m), seamount (50 m), seamount (250 m), seamount (500 m)	Used	This study	Method

Supplementary Section S2: Imputing missing length data on BRUVS

Due to the random nature of fish behavior, all individually counted fish could not be measured. When possible, each species on each BRUVS was measured up to its abundance level to a limit of ten individuals to optimize processing time. A total of 3369 individual measures were used, from 281 out the 443 (63%) species. Averaging the individual measures per species per BRUVS yielded 1315 averaged lengths. 2009 remaining lengths of species on different BRUVS were therefore imputed using the random forest algorithm and the *missForest v1.4* R (Stekhoven & Buhlmann 2012) package with 999 trees. Lengths were estimated based on measured lengths when the species presented other records, but also family, genus, and maximum size and size type from FishBase (https://www.fishbase.se). Latitude and longitude of available lengths of species were also used to account for geographic proximity of existing lengths. The missForest accuracy was tested with a k-fold cross validation procedure by predicting 5% of the lengths each time by training the missForest on the 95% left of the data and look at the linear fit between the original and predicted value. Results showed a mean R^2 of 0.82 (SD ± 0.07) between the measured and predicted lengths of the missForest model (Fig. S2).

As we did not seek highly accurate length structure among replicates but rather the general pattern of lengths and then biomass between assemblages among our stratum, we considered the imputed values exploitable with an average r^2 of 0.84. We also ensured that imputed length did not exceed Fishbase's max reported length.

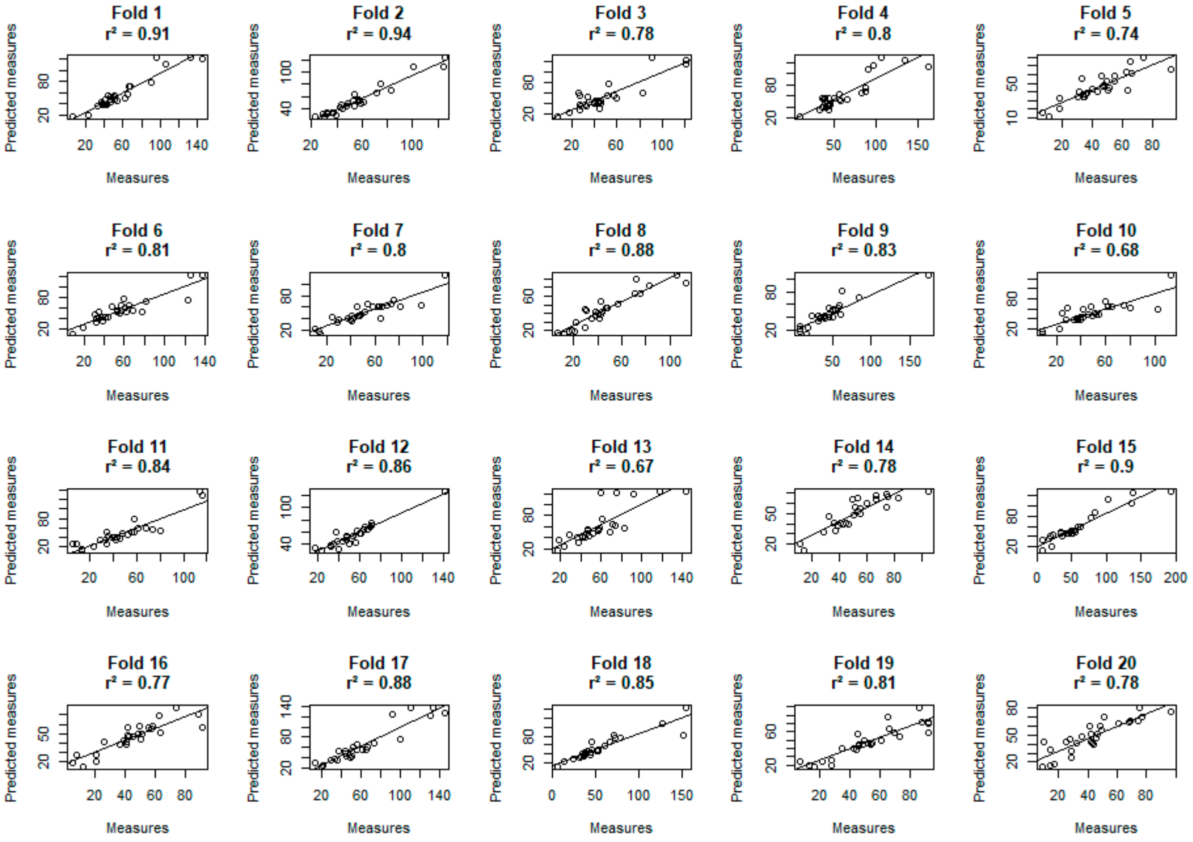

Figure S1: Predicted against true measures of fish for each of the twenty-fold cross-validation results from the 5% artificial imputation using missForest out of the 792-measurement data.

Supplementary Section S3: Boosted regression trees on richness, biomass and biomass of large predators

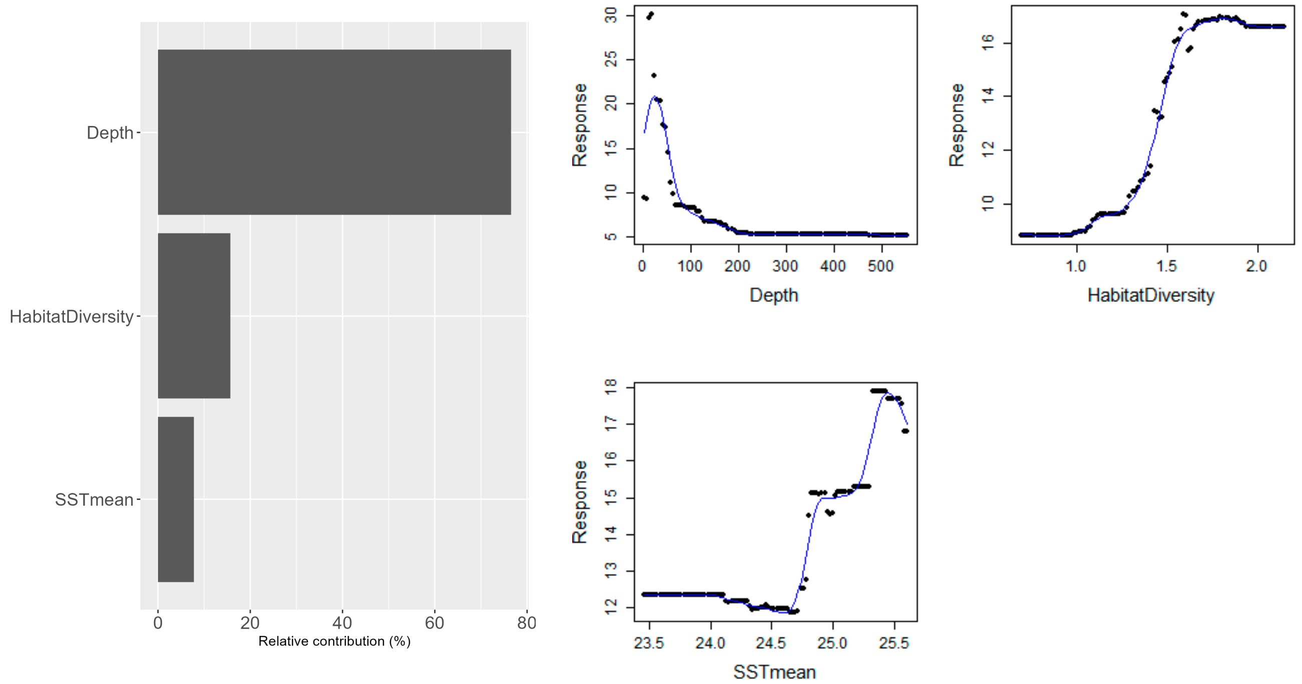

Figure S2: Variable importance and partial dependance plots from boosted regression trees ran on the reduced number of variables on BRUVS data with richness as the predicted value (response). Cross validation correlation value was 0.89 for this model.

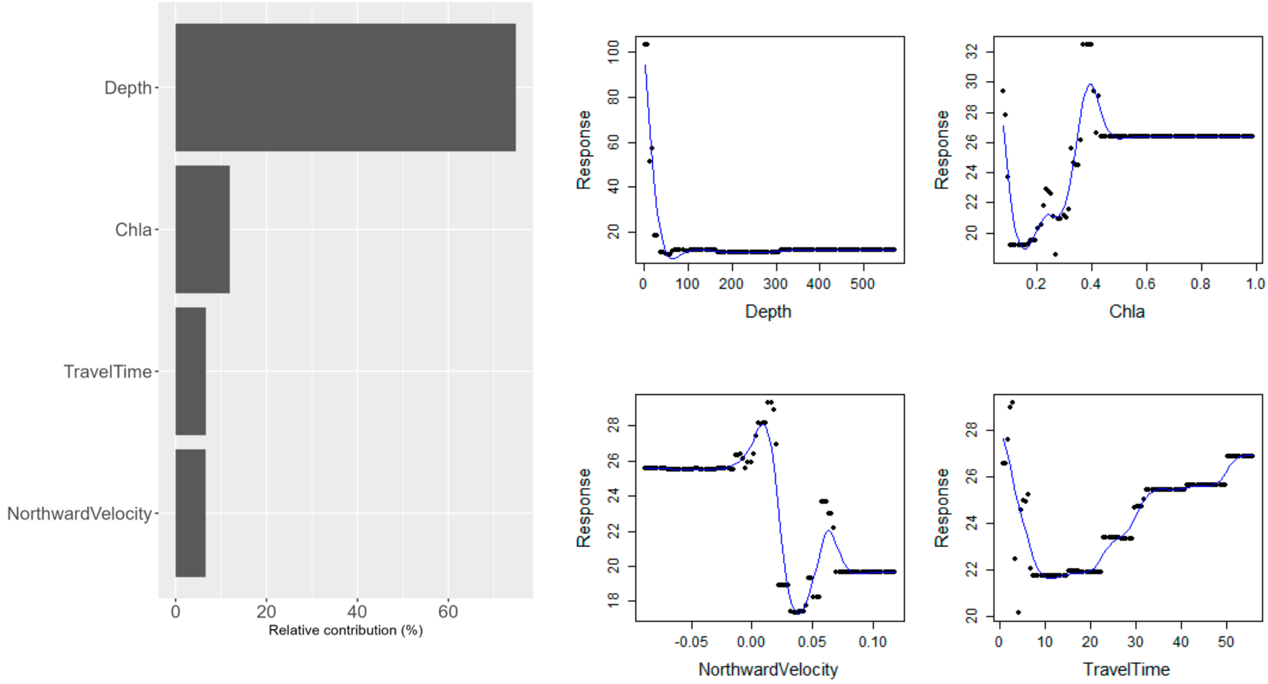

Figure S3: Variable importance and partial dependance plots from boosted regression trees ran on the reduced number of variables through previous boosted trees run on eDNA data with MOTU richness as the predicted value (response). Cross validation correlation value was 0.86 for this model.

Figure S4: Variable importance and partial dependance plots from boosted regression trees ran on the reduced number of variables through previous boosted trees run on BRUVS data with biomass as the predicted value (response). Cross validation correlation value was 0.71 for this model

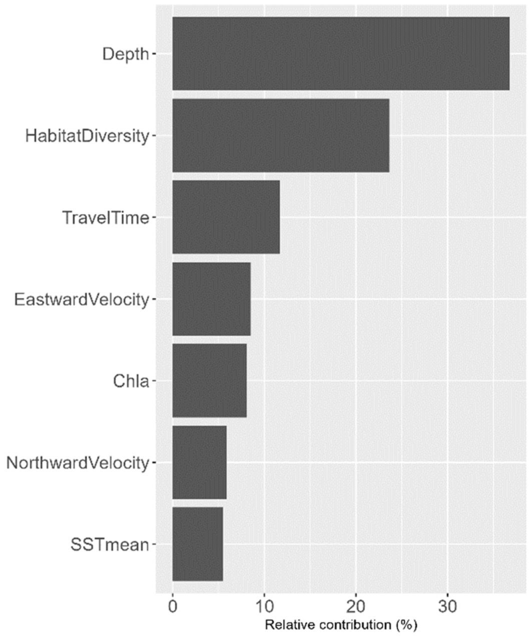

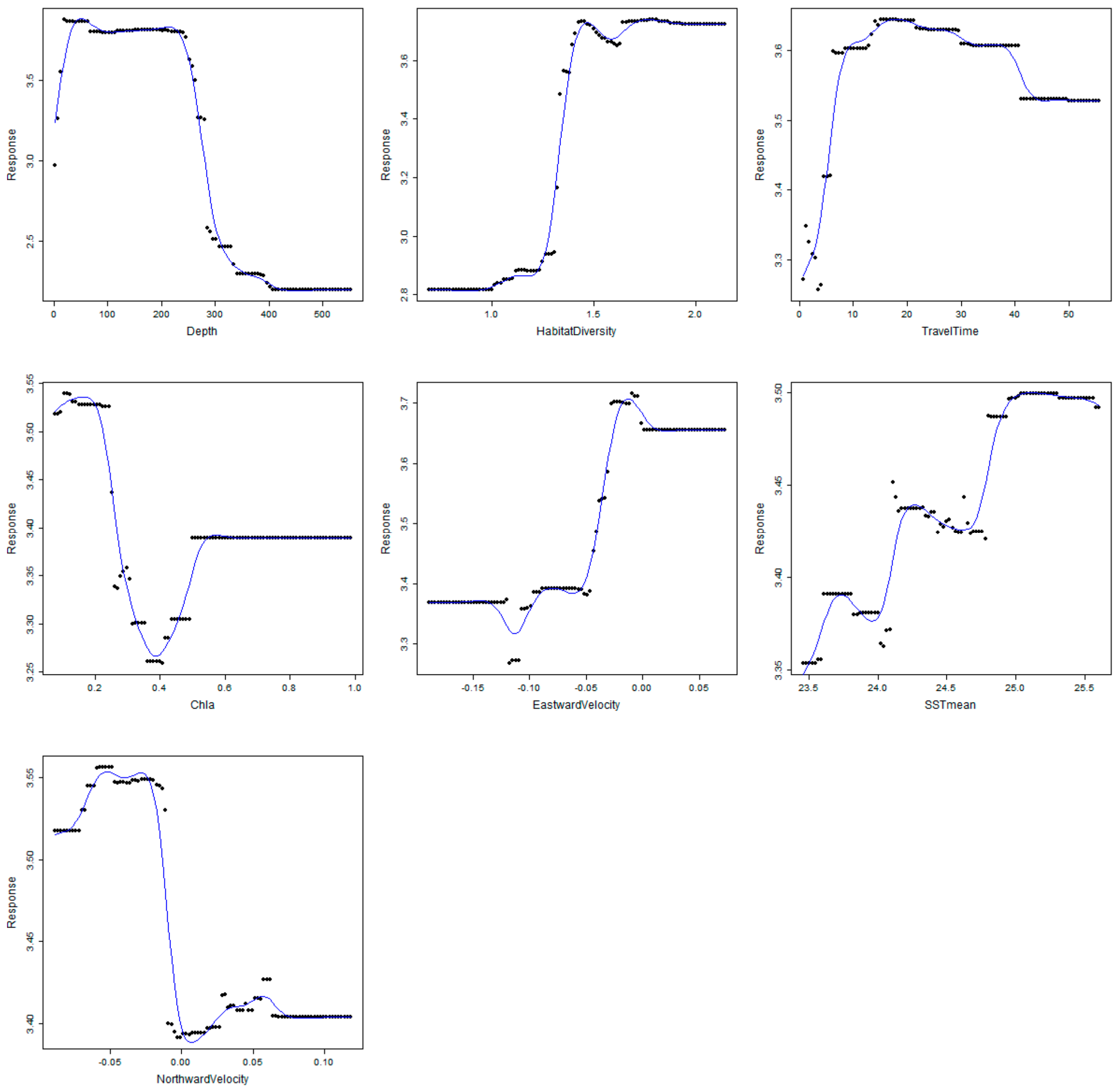

Figure S5: Variable importance and partial dependance plots from boosted regression trees ran on the reduced number of variables through previous boosted trees run on BRUVS data with biomass of large predators as the predicted value (response). Cross validation correlation value was 0.75 for this model.

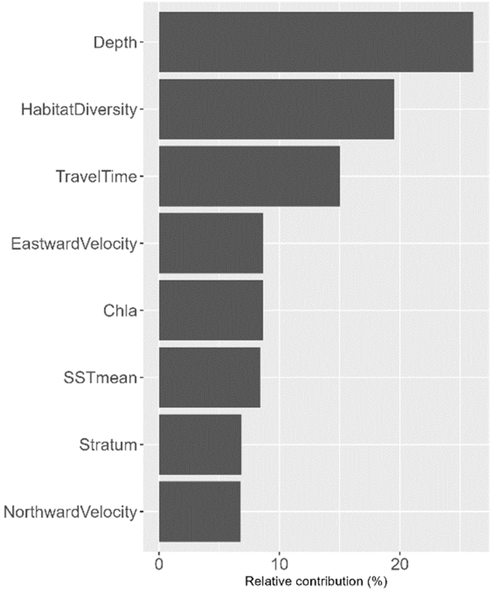

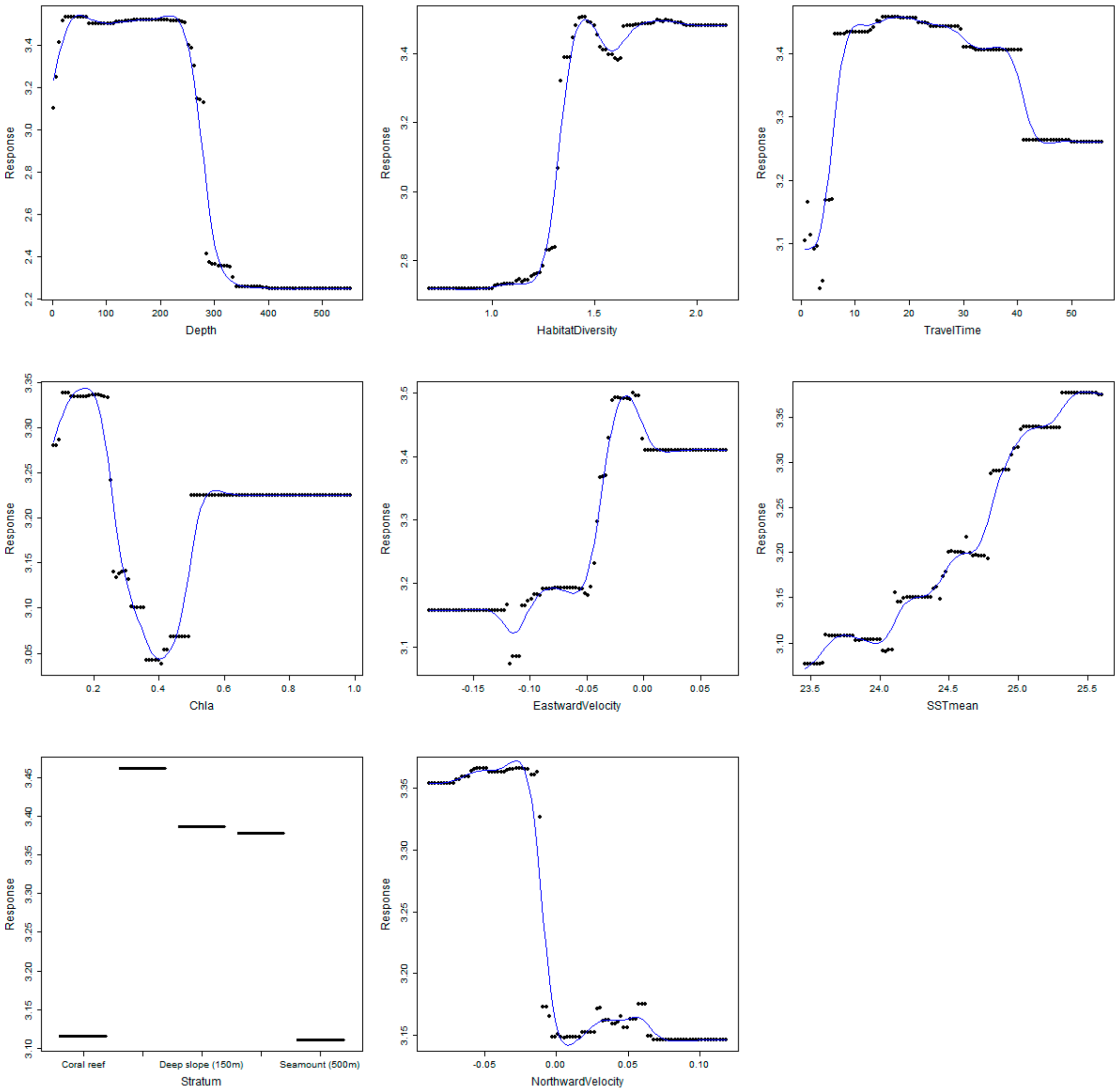

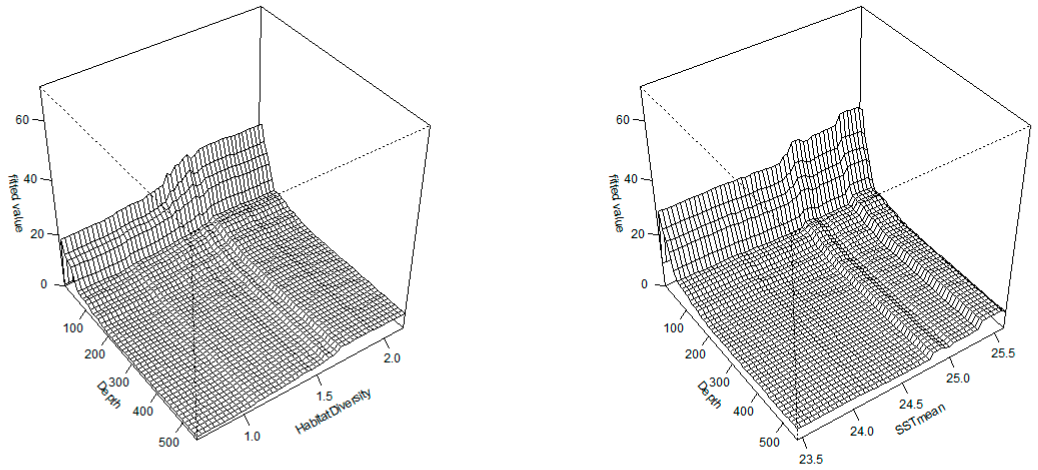

Figure S6: Interaction plots found from boosted regression trees ran on the reduced number of variables on BRUVS data with richness as the predicted value (fitted value). Cross validation correlation value was 0.89 for this model.

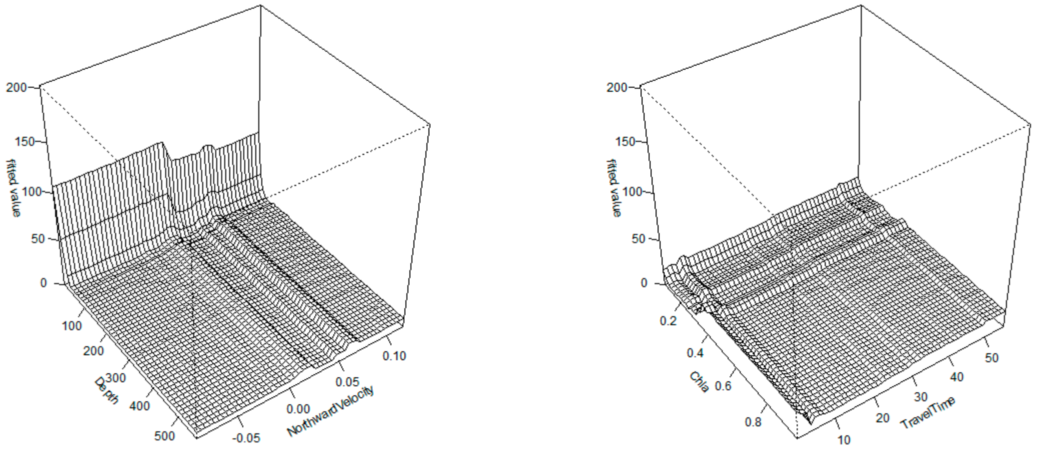

Figure S7: Interaction plots found from boosted regression trees ran on the reduced number of variables through previous boosted trees run on eDNA data with MOTU richness as the predicted value (response). Cross validation correlation value was 0.86 for this model.

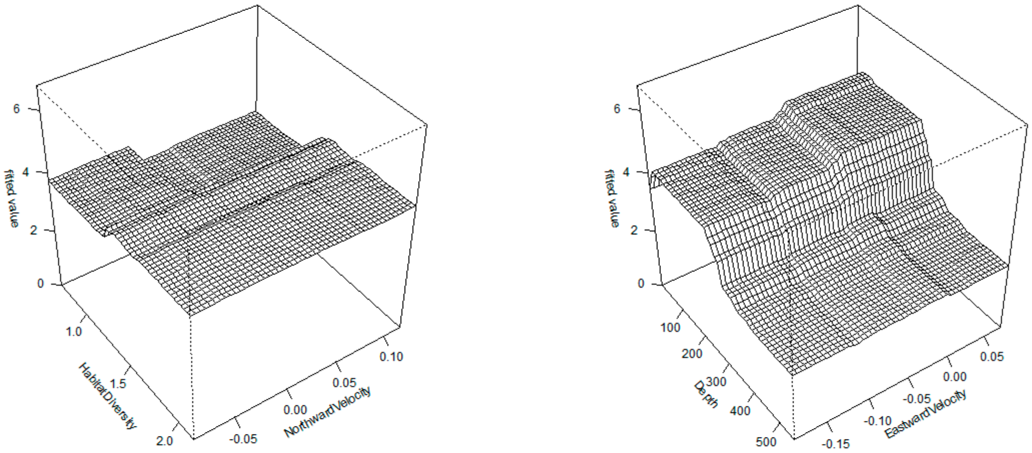

Figure S8: Interaction plots found from boosted regression trees ran on the reduced number of variables through previous boosted trees run on BRUVS data with biomass as the predicted value (response). Cross validation correlation value was 0.71 for this model.

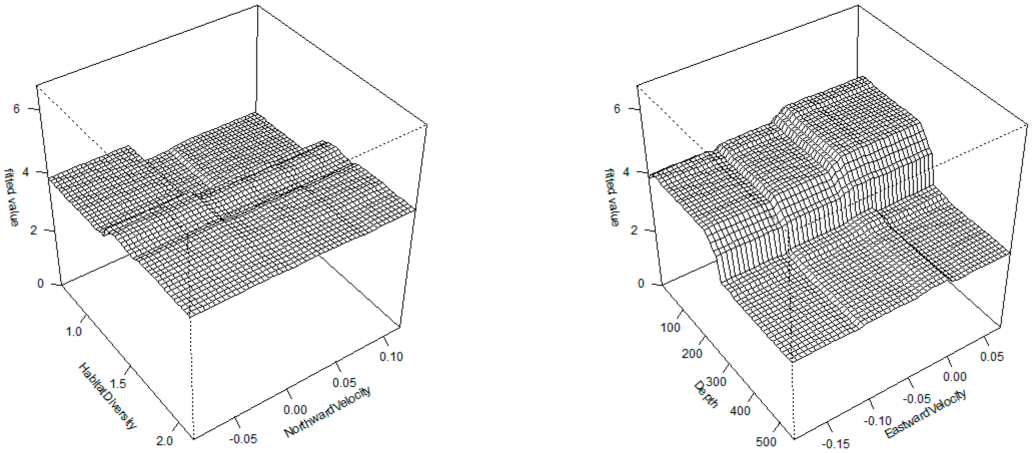

Figure S9: Interaction plots found from boosted regression trees ran on the reduced number of variables through previous boosted trees run on BRUVS data with biomass of large predators as the predicted value (response). Cross validation correlation value was 0.75 for this model.

Supplementary Section S4: Assemblage ordination

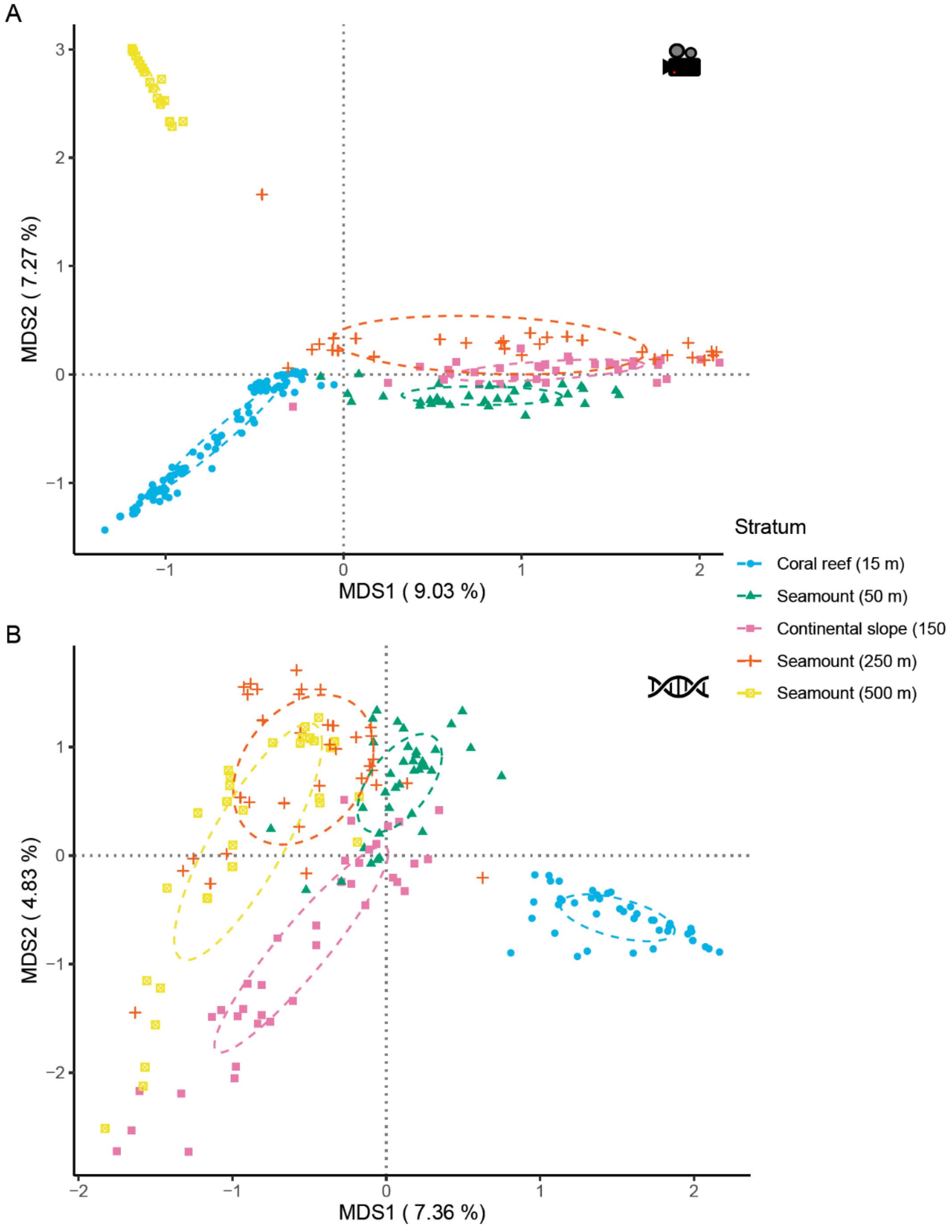

Figure S10: Ordination using Principal Coordinate Analysis (PCoA) on the Hellinger transformed distance matrix of baited remote underwater video stations (BRUVS) abundance data (A) and presence-absence environmental DNA (eDNA) data (B). Ellipses were calculated from the covavariance matrix of each stratum weighted by the number of points.

Supplementary Section S5: sharing of eDNA sequences among the environmental strata

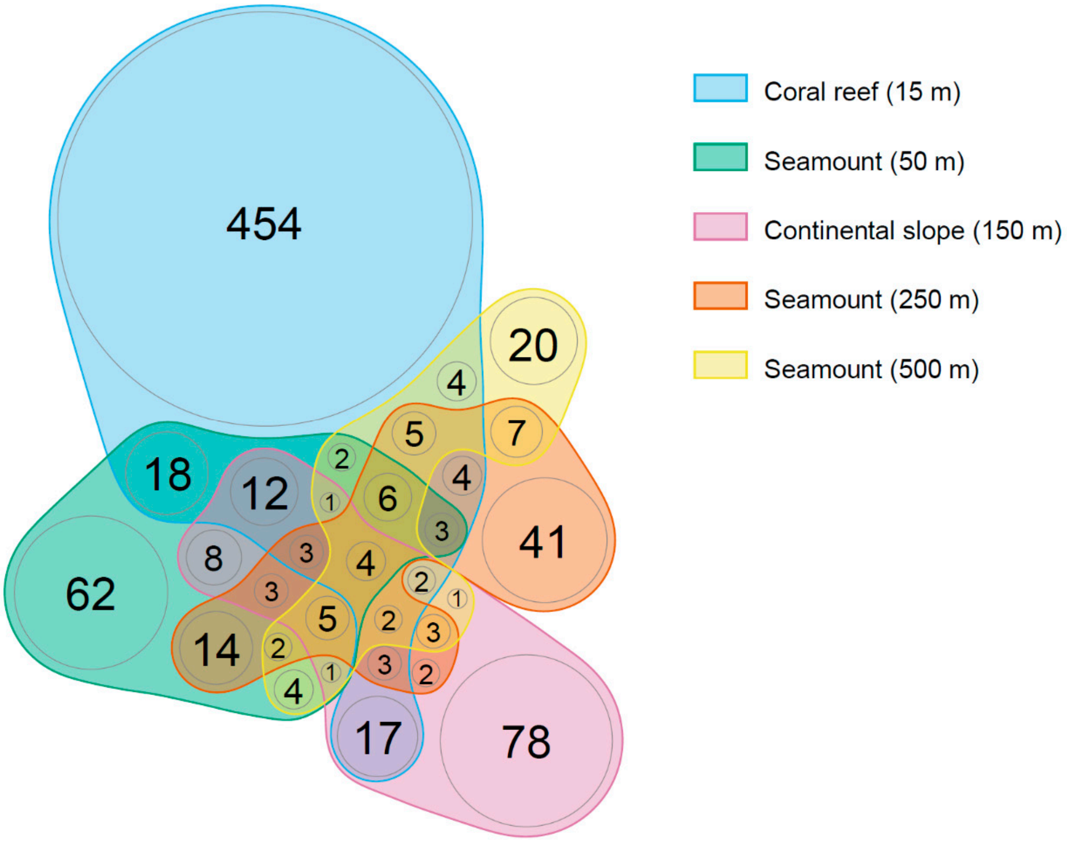

Figure S11: Euler diagram of identified MOTUs from eDNA metabarcoding along 5 environmental strata: coral reefs, seamounts of variable summit depths and continental slopes.

References

Baletaud F, Gilbert A, Mouillot D, Come J-M, Vigliola L. 2022. Baited video reveal fish diversity in the vast inter-reef habitats of a marine tropical lagoon. Marine Biodiversity **52**:16. Available from https://link.springer.com/10.1007/s12526-021-01251-3.

Barletta M, Barletta-Bergan A, Saint-Paul U, Hubold G. 2005. The role of salinity in structuring the fish assemblages in a tropical estuary. Journal of Fish Biology **66**:45–72. Available from https://onlinelibrary.wiley.com/doi/10.1111/j.0022-1112.2005.00582.x.

Boettiger C, Lang DT, Wainwright PC. 2012. rfishbase: exploring, manipulating and visualizing FishBase data from R. Journal of Fish Biology **81**:2030–2039. Wiley-Blackwell.

Capblancq J. 1990. Nutrient dynamics and pelagic food web interactions in oligotrophic and eutrophic environments: an overview. Hydrobiologia **207**:1–14. Available from http://link.springer.com/10.1007/BF00041435.

Eduardo LN, Frédou T, Lira AS, Ferreira BP, Bertrand A, Ménard F, Frédou FL. 2018. Identifying key habitat and spatial patterns of fish biodiversity in the tropical Brazilian continental shelf. Continental Shelf Research **166**:108–118. Elsevier Ltd. Available from https://doi.org/10.1016/j.csr.2018.07.002.

Leitner AB, Neuheimer AB, Drazen JC. 2020. Evidence for long-term seamount-induced chlorophyll enhancements. Scientific Reports **10**:1–10. Nature Publishing Group UK. Available from https://doi.org/10.1038/s41598-020-69564-0.

Letessier TB et al. 2019. Remote reefs and seamounts are the last refuges for marine predators across the Indo-Pacific. PLOS Biology **17**:e3000366. Available from https://dx.plos.org/10.1371/journal.pbio.3000489.

Letessier TB, Juhel JB, Vigliola L, Meeuwig JJ. 2015. Low-cost small action cameras in stereo generates accurate underwater measurements of fish. Journal of Experimental Marine Biology and Ecology **466**:120–126. Elsevier B.V. Available from http://dx.doi.org/10.1016/j.jembe.2015.02.013.

Maire E, Cinner J, Velez L, Huchery C, Mora C, Dagata S, Vigliola L, Wantiez L, Kulbicki M, Mouillot D. 2016. How accessible are coral reefs to people? A global assessment based on travel time. Ecology Letters **19**:351–360. Available from http://doi.wiley.com/10.1111/ele.12577.

Priede IG et al. 2013. Does Presence of a Mid-Ocean Ridge Enhance Biomass and Biodiversity? PLoS ONE **8**.

Rogers AD. 2018. The Biology of Seamounts: 25 Years on. Pages 137–224 Advances in Marine Biology. Available from https://linkinghub.elsevier.com/retrieve/pii/S0065288118300038.

Sih T, Daniell J, Bridge T, Beaman R, Cappo M, Kingsford M. 2019. Deep-Reef Fish Communities of the Great Barrier Reef Shelf-Break: Trophic Structure and Habitat Associations. Diversity **11**:26. Available from http://www.mdpi.com/1424-2818/11/2/26.

Stekhoven DJ, Buhlmann P. 2012. MissForest--non-parametric missing value imputation for mixed-type data. Bioinformatics **28**:112–118. Available from https://academic.oup.com/bioinformatics/article-lookup/doi/10.1093/bioinformatics/btr597.

Tittensor DP, Mora C, Jetz W, Lotze HK, Ricard D, Berghe E vanden, Worm B. 2010. Global patterns and predictors of marine biodiversity across taxa. Nature **466**:1098–1101.

Woolley SNC, Tittensor DP, Dunstan PK, Guillera-Arroita G, Lahoz-Monfort JJ, Wintle BA, Worm B, O'Hara TD. 2016. Deep-sea diversity patterns are shaped by energy availability. Nature **533**:393–396. Nature Publishing Group. Available from http://dx.doi.org/10.1038/nature17937.
